# Research and Optimization of Extrusion Tap Structure Based on Numerical Simulation and Experimental Analysis

**DOI:** 10.3390/ma17081891

**Published:** 2024-04-19

**Authors:** Yi Tao, Nian Wan, Daoming Xu, Qiang He

**Affiliations:** 1 Marine Design & Research Institute of China, Shanghai 200011, China; 2 School of Mechanical Engineering, Jiangsu University of Science and Technology, Zhenjiang 212000, China

**Keywords:** internal thread, extrusion forming, tap structure, parameter optimization, numerical simulation

## Abstract

In order to enhance the quality of thread processing by tap, a systematic analysis of its forming mechanism and factors affecting forming quality is conducted. Effects of the number of edges, the amount of shovel back, the extrusion cone, the calibration part and the extrusion cone angle on the torque and temperature are achieved by finite element analysis and experiments. From the perspective of reducing torque and temperature during the forming process, the optimal combination of tap structural parameters for machining M22×2 internal threads on 42CrMo4 high-strength steel are further obtained through orthogonal optimization. The results show that, unlike the cutting process of threads, the extrusion forming process of threads is a net forming process in which metal undergoes plastic deformation in a limited space, and the metal material continuously flows along the edge of the V-shaped groove of the tap, gradually piling up to form the thread tooth shape. This also caused a noticeable lack of flesh at the top of the extruded thread teeth. Better quality threads can be obtained by machining with optimized structural parameters. The maximum torque and temperature during the machining process are reduced by 22.86% and 20.31%, respectively. The depth of the hardened layer increased by 0.05 mm, and the root and top hardness of the teeth increased by 10 HV_0.2_ and 5 HV_0.2_, respectively.

## 1. Introduction

With the rapid development of modern mechanical manufacturing industry, the design requirements of mechanical equipment are becoming more stringent. The reliability and stability of performance are important indexes to evaluate the quality of equipment. Thread connection is widely used in the connection and fixation of various mechanical parts [1]. When the mechanical equipment is in operation, the part of the thread connection will bear larger radial, axial and shear loads. Therefore, the influence of thread forming quality on the service performance of mechanical equipment is crucial [2,3,4]. 

Traditional threads are mainly formed through cutting processing, and many researchers have studied the machining process of cutting tap. Cao et al. [5,6] predicted the torque and axial force in the machining process by establishing a mechanical model, and verified the accuracy of the model with tests. To reduce the torque during the cutting process, Filho et al. [7] analyzed the influence of different cooling lubricants on tapping through experiments. Biermann et al. [8,9,10] optimized the structure of spiral groove of cutting tap through the method of fluid simulation, thus reducing the temperature in the tapping process and improving the quality of the cutting thread. 

To evaluate the wear of tap during the cutting process, Xu et al. [11,12] established a deep learning-based model system for the monitoring and prediction of tool wearing. Korhonen et al. [13,14,15] adopted the method of adding coating to the surface of cutting tap and reducing the wear of the tap, thus prolonging its service life. Hou et al. [16,17] added low-frequency vibration to the tapping process. To maximize the efficiency of low-frequency vibration, Paulsen et al. [18,19] analyzed the relationship between the optimum amplitude and the material properties of workpieces through experiments. Pereira et al. [20,21,22] researched the torque and temperature during the cutting process. 

Changing the tool from a cutting tap to an extrusion tap is an effective method to improve the quality of thread forming. The difference is that the thread extrusion forming process is a method that utilizes the sharp teeth of the tap to force the metal in the deformation zone around the workpiece hole wall to flow plastic along the V-shaped groove, thereby forming a thread shape. Badami et al. [23,24,25] found that the average tensile load of extruded threads is about 20% higher than that of cutting threads through tensile experiments. Liu et al. [26] determined that the hardness of extruded threads is higher than that of cutting threads through hardness measurement experiments. Wittke et al. [27] found that the number of fatigue cycles of extruded threads is more than that of cutting threads through fatigue experiments. Through the research of scientific researchers, it has been found that the quality of internal threads formed by the cold extrusion process is better [28,29,30].

However, most existing research concentrates on the effect of processing methods and processing parameters on thread quality, while there is little research on the influence of the structure of the extruded tap on thread quality. When the machining environment, such as workpiece material, thread specification and process parameters, changes, the forming quality of thread will be affected. So, it is necessary to optimize the structure of tap according to the processing environment of the enterprise.

In this paper, the working mechanism of tap is overviewed by theoretical analysis. The torque and temperature during tap machining are monitored through experiments and numerical simulations. The forming quality of thread is analyzed, from the point of view of tooth height, surface microstructure, hardness and hardened layer, by using the shape measurement laser microscope system and the automatic micro-Vickers hardness measurement system. Based on the analysis of the influences of structural parameters, the structure of the tap is further optimized. The thread quality, before and after optimization, is analyzed from the angle of tooth height, hardness and hardened layer, so as to evaluate the optimization of the optimized tap.

## 2. Working Mechanism of Tap

**Figure 1:**
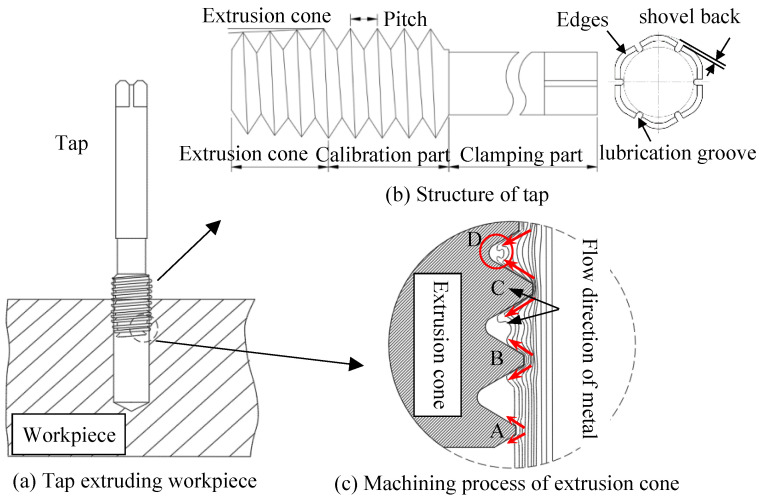
Machining process of extruded tap [31].

The quiddity of machining thread by tap is that the metal of the workpiece has plastic deformation under the extrusion of tapered teeth. The metal flows along the tapered teeth, gradually filling the grooves between the teeth, and finally forming the thread. The tapping process is shown in Figure 1a. When the tap starts working, a tooth begins to extrude the workpiece and the metal runs along both sides of a tooth. The tooth moves forward by one pitch for each rotation, and the structure of the tap is shown in Figure 1b. 

Figure 1c shows the machining process. The depth of C tooth extrusion is further increased, and the tooth profile between the B tooth and C tooth will rise due to the inflow of more metal, initially forming the outline of the thread. As the processing continues, the depth of the D tooth extrusion is further increased, and the groove between the C tooth and D tooth will form a more complete tooth profile. Subsequently, the calibration part begins to squeeze. The function of the calibration section is to reform the preliminarily formed thread tooth profile. When the last tooth of calibration part leaves the extruded area, the metal in the area will stand firmly in the form of teeth. Finally, the thread that meets the size and shape requirements is formed.

## 3. Tap machining Experiment

### 3.1. Construction of Experimental Platform

#### 3.1.1. Extruded Tap and Workpiece

**Figure 2:**
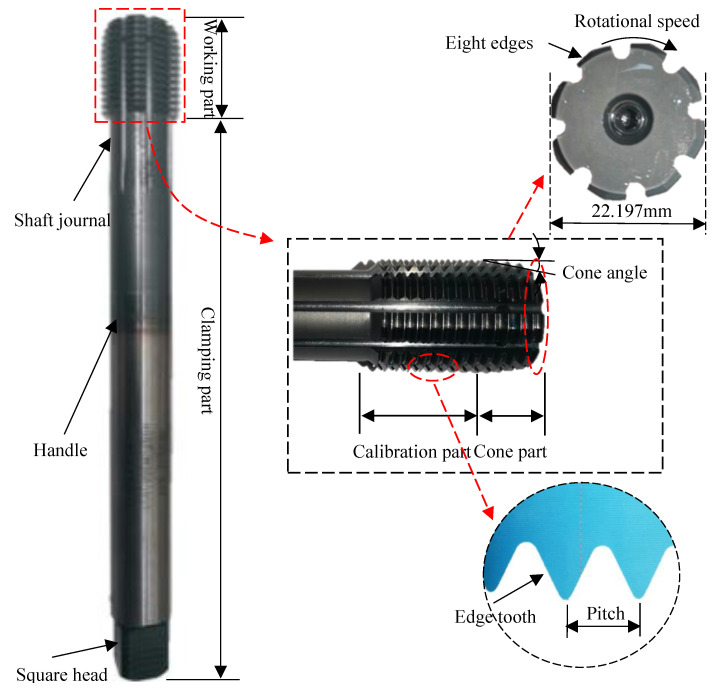
Structure of extruded tap.

To study the quality of thread before optimization, the experiment of thread machining is conducted on the horizontal machining center. In Figure 2, the tap consists of a working part (including extrusion cone and calibration component) and a clamping part. The specific parameters of tap are as follows: extrusion cone angle 6°, length of extrusion part 8 mm, length of calibration 18 mm, number of edges 8, amount of shovel back 0.6 mm, and pitch 2 mm. The clamping part is composed of a journal, a knife handle and a square head. The clamping part is only used for the clamping and fixing of the tap, which almost has no effect on the quality of the thread. The workpiece used in the test is a square block of 42CrMo4 steel, with the workpiece size 50 mm× 50 mm× 20 mm and through-hole size ø21.20 mm. The chemical composition and mechanical properties are shown in Table 1 and Table 2, respectively.

**Figure 3:**
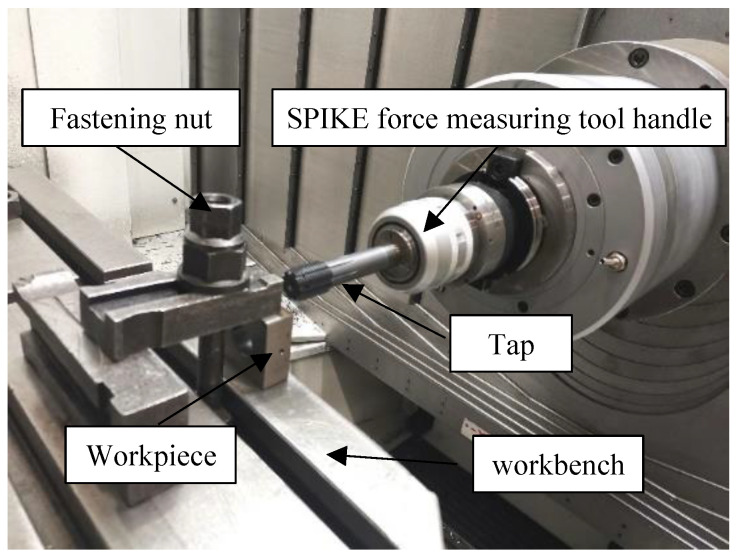
Thread processing.

Before the test, the remaining metal chips and other residues on the workbench should be cleaned. Then, the square workpiece should be placed vertically on the workbench, the square workpiece placed vertically on the table, and the workpiece firmly fixed between the platen and the table by rotating the tightening nut. The tap is connected to the SPIKE force measuring tool handle using a variable diameter sleeve and fixed on the tail seat of the machine tool. As the tap rotates and feeds, threads are machined on the inner wall of the workpiece through the hole, as shown in Figure 3.

#### 3.1.2. Measurement of Extrusion Torque and Temperature

**Figure 4:**
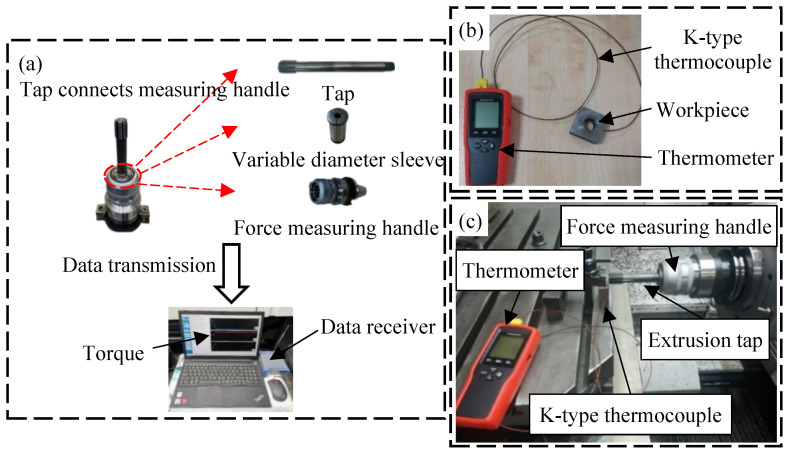
Experimental preparation: (**a**) Measurement of extrusion torque; (**b**) Measurement of extrusion temperature; (**c**) Thread machining experiment.

The tap and force measuring handle are connected by a variable diameter sleeve, and the data of torque is transmitted to a computer through the data receiver, as shown in Figure 4a. Regarding the measurement of the extrusion temperature, since the deformation zone is located at the through hole of the workpiece, the temperature of the deformation zone is hard to measure directly. Therefore, the extrusion temperature is measured by drilling a hole on the outer surface of the workpiece. To reduce the error in measuring, there is a need to make the hole as close to the deformation zone as possible, so the depth of drilling is 12 mm. The size of the hole is a little bigger than that of the thermocouple. K-type thermocouple is slotted into the hole to perceive temperature change in the deformation zone. And the thermocouple is linked with the thermometer to store the data, as shown in Figure 4b. The overall situation of the experiment is shown in Figure 4c.

### 3.2. Result of Experiment

#### 3.2.1. Analysis of Extrusion Torque and Temperature

**Figure 5:**
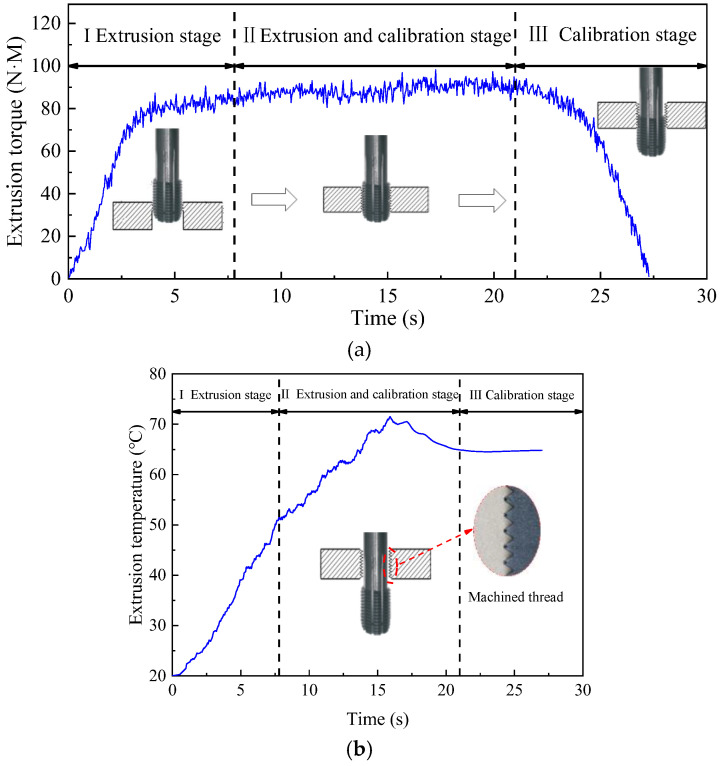
Extrusion torque (**a**) and temperature (**b**) in machining process.

Figure 5a,b shows the experimental curve, which consists of three stages. First, the extrusion cone of tap begins to process the workpiece, with the torque and temperature increasing sharply at this stage. In the second stage, with the further processing of tap, the calibration part begins to squeeze into the workpiece. In this stage, the extrusion cone and the calibration part extrude the metal together. The torque and temperature continue to increase to the maximum value. The maximum extrusion torque and temperature are 98.06 N·M and 71.49 °C, respectively. However, since there is no cone angle in the calibration part, the increase in torque and temperature is smaller than that in the first stage. In the third stage, the extrusion cone leaves the workpiece, and only the calibration department is processing the workpiece. As the calibration part leaves the workpiece, the torque and temperature gradually decrease. When the calibration part completes the machining of the workpiece, the metal at the through hole of the workpiece will stand firmly in the form of teeth.

#### 3.2.2. Analysis of Microstructure

**Figure 6:**
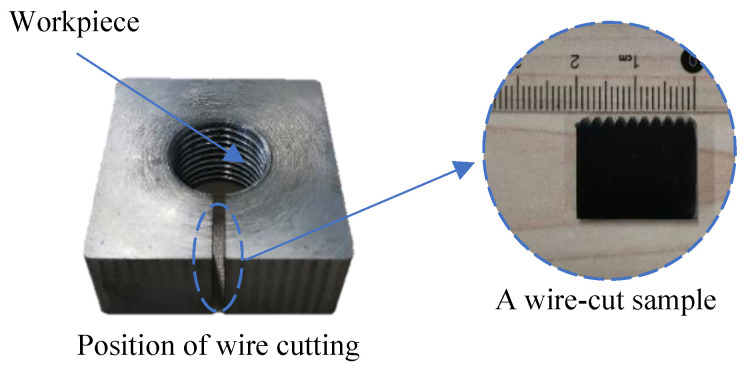
Thread sample [31].

The thread is analyzed from the point of view of microstructure. A small sample is obtained through the use of the wire-cutting device, as shown in Figure 6. The threaded sample is eroded with 4% nitric acid alcohol solution. Through the laser microscope system, the microstructure of thread is studied from three parts: tooth top, tooth side and tooth root.

**Figure 7:**
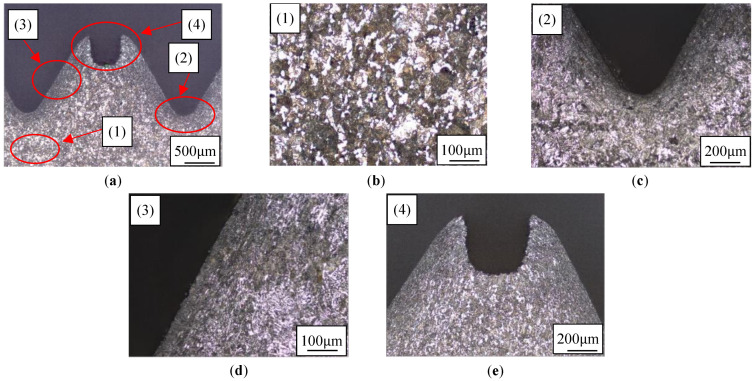
Microstructure of thread. (**a**) Thread; (**b**) Original material; (**c**) Tooth root; (**d**) Tooth side; (**e**) Tooth top.

As shown in Figure 7a, the microstructure of metal located at the threaded has undergone obvious changes. The metal undergoes plastic deformation under the extrusion action. Grains are warped and stretched into a streamlined fiber structure. So, the degree of refinement of fibrous structure inside the thread is significantly higher, while the metal far away from the thread area is almost not squeezed by the tap. There is no obvious deformation of the metal in this area. The extent of grain refinement of fiber structure is not changed, as shown in Figure 7b.

It is precisely the difference in extrusion pressure that leads to differences in microstructure. Tooth root is directly contacted with the tap diameter, which bears the greatest extrusion load, and therefore grain refinement of the metal fiber structure is the most obvious. The metal fiber tissue at tooth root is severely squeezed, to the extent that it is unable to distinguish the grains, forming a curved streamline that flows toward the tooth side, as shown in Figure 7c. The metal fiber structure on the tooth side forms a straight line approximately parallel to the side wall, as shown in Figure 7d. The metal fibrous structure at the tooth top is sparse, as shown in Figure 7e.

#### 3.2.3. Measurement of Hardness and Hardened Layer

**Figure 8:**
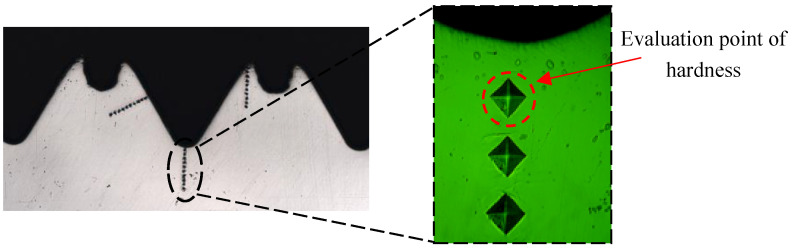
Measuring point of hardness [31].

For measurement, the hardness tester sets the load on the thread and the holding load time to 200 g and 15 s, respectively. Figure 8 shows the selected positions of measurement points. The nearest measuring point on the thread to the surface is used to evaluate the hardness. 

**Figure 9:**
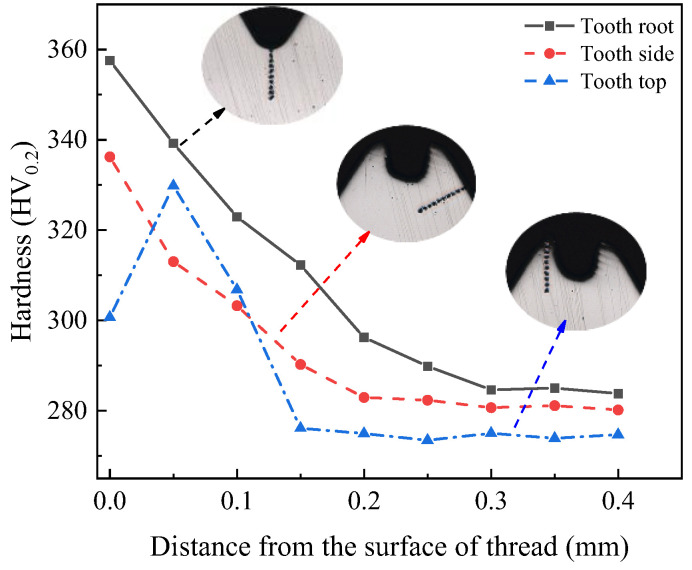
Measurement of thread hardness [31].

Figure 9 shows the measurement results, demonstrating that the hardness varies significantly in different positions, which is because these parts have undergone varying degrees of plastic deformation. While the extent of metal deformation will directly affect the work hardening, the maximum hardness and hardening depth at the tooth root are 367.74 HV_0.2_ and 0.3 mm, respectively. The surface hardness gradually decreases as the distance from the thread surface increases, which is because the extrusion pressure on the metal decreases as the depth increases. As the degree of deformation decreases, the extent of grain refinement of fiber structure decreases, and so the increase in hardness gradually decreases until it returns to the hardness of the original material. Compared with tooth root, the degree of plastic deformation on the tooth side decreases. The hardness and depth of the hardened layer also decrease. The minimum hardness and hardening depth at the tooth tip are 300.69 HV_0.2_ and 0.15 mm, respectively.

## 4. Numerical Simulation of Tap Machining

### 4.1. Establishment and Validation of Model

**Figure 10:**
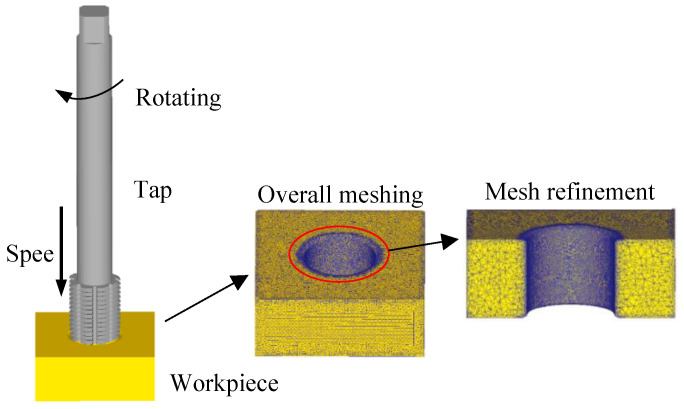
Finite element model of tap and workpiece [31].

The models of tap with different structures are first established in the Solidworks2022, and then the taps are changed into stl format and leading-in DEFORM-3D. As shown in Figure 10, the tap is set as a rigid body without considering wear of the tap. The workpiece is set as a plastic body, the elastic recovery is not considered, and the material is set to 42CrMo4. The local mesh refinement is carried out at the through hole of the workpiece, with a total of 115,007 nodes and 529,281 elements. The motion parameters of tap are set based on the relationship between pitch, feed rate and tool speed. The rotation speed is 50 r/min and the feed speed is 1.667 mm/s. The friction coefficient is set to 0.20. As the motion distance of tap reaches 50 mm, the simulation stops. The number and size of steps are set to 650 and 0.08 mm, respectively.

**Figure 11:**
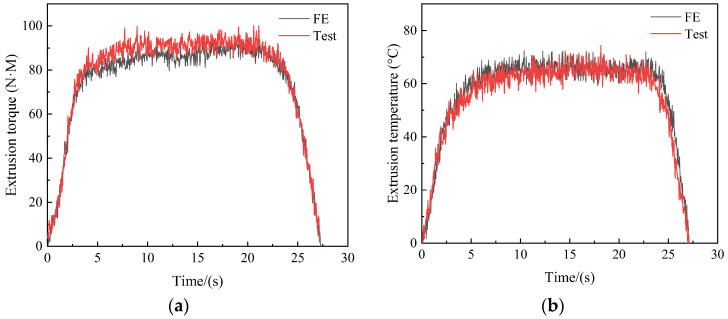
Comparison between numerical simulation and experiment: (**a**) Extrusion torque, (**b**) Extrusion temperature.

The comparison of extrusion torque and temperature between the experiment and simulation are shown in Figure 11a,b. The maximum extrusion torque measured in actual experiments is 101.95 N·M, while the maximum extrusion torque obtained in numerical simulations is 98.98 N·M, with an error of only 2.91% between the two; The maximum extrusion temperature measured was 57.19 °C, and the maximum extrusion temperature obtained from numerical simulation was 54.95 °C, with an error of 3.92%. This result verifies the accuracy of the finite element model for extrusion taps.

### 4.2. Effect of Tap Structure on Extrusion Process

**Figure 12:**
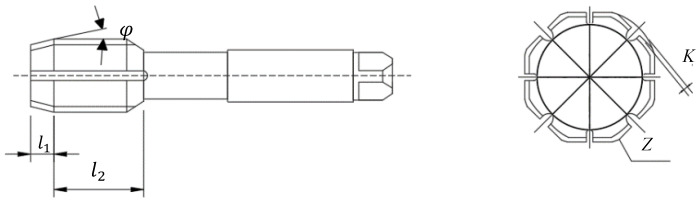
Structure of extrusion tap.

Combined with the design experience of the enterprise, the key parameters of the tap are as follows: the number of edges Z, shovel back amount K, extrusion cone angle φ, extrusion cone $l_{1}$ and calibration part $l_{2}$ are optimized. Figure 12 presents the structure of the extrusion tap.

#### 4.2.1. Analysis of Number of Edges

**Figure 13:**
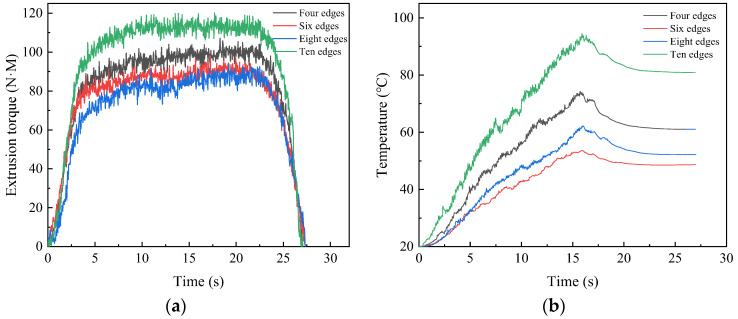
Extrusion torque (**a**) and temperature (**b**) under different edges.

For tap with a specification of M22, the common number of edges is an even number of more than four. The simulation is conducted with number of edges four, six, eight and ten, respectively. So as to study the effect of edge number on extrusion torque and temperature, Table 3 shows the parameters used in the experiment and numerical simulation. The curves under different edges are shown in Figure 13a,b. 

When the number of edges is four, the maximum torque and temperature are 107.13 N·M and 74.30 °C, respectively. As the number of edges increased to eight, the torque decreased to 93.88 N·M and the temperature decreased to 62.15 °C. The increase of edges makes the section of tap approaches into a circle from a curved edge, which reduces the force required for the tap to rotate, thus reducing the torque. The quasi-circular section increases the stationarity of tap in the machining process, which reduces the friction and thereby lowers the temperature. When the number of edges continues to increase to 10, the torque and temperature continue to increase. This is due to the fact that when there are too many edges, the contact area increases. The amount of metal that needs to be extruded increases rapidly, which promotes an increase in torque and temperature.

#### 4.2.2. Analysis of Shovel Back

**Figure 14:**
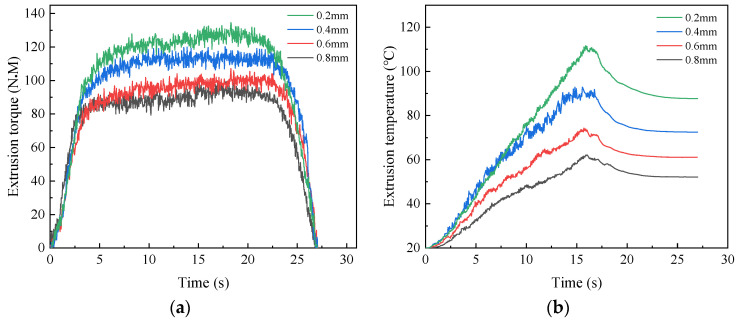
Extrusion torque (**a**) and temperature (**b**) under different shovel back.

The value of shovel back is affected by many factors. According to the production experience of enterprise, the numerical simulation was conducted with the amount of shovel back 0.2 mm, 0.4 mm, 0.6 mm and 0.8 mm. Curves under different shovel backs are shown in Figure 14a,b. 

When the amount of shovel back is 0.2 mm, the maximum torque and temperature are 134.53 N·M and 111.61 °C, respectively. As amount of shovel back increased to 0.8 mm, the torque and temperature decreased to 99.30 N·M and 62.15 °C, respectively. This is due to the fact that the increase of shovel back will, on the one hand, cause the ridge of tap to be sharp, so the contact surface between the ridge and the workpiece will be reduced. On the other hand, the increase of shovel back will result in the decrease of the arc radius of tap, reducing the contact surface of the workpiece. The friction between tap and workpiece is further reduced, resulting in a reduction in torque and temperature.

#### 4.2.3. Analysis of Extrusion Cone

**Figure 15:**
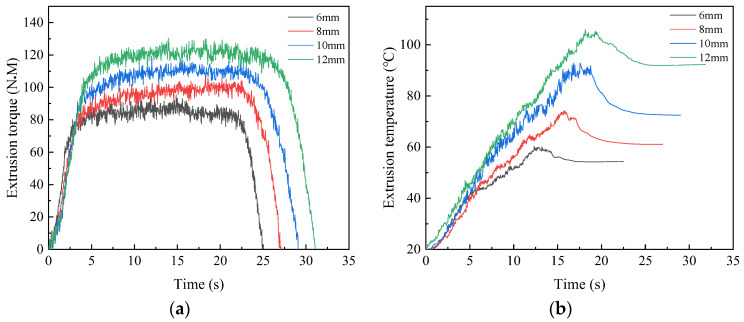
Extrusion torque (**a**) and temperature (**b**) under different extrusion cone.

The tap models with different extrusion cone were established for numerical simulation. The length of extrusion cone is generally three to six pitchs, and the numerical simulation is therefore conducted with the extrusion cone 6 mm, 8 mm, 10 mm and 12 mm. The curves under different extrusion cones are shown in Figure 15a,b.

When the length of the extrusion cone is 6mm, the maximum torque and temperature are 95.37 N·M and 60.30 °C, respectively. As length of extrusion cone increases to 12 mm, the maximum torque and temperature increase to 130.38 N·M and 105.83 °C, respectively. As the length of extrusion cone increases, the contact surface area of workpiece increases. This leads an increase in the amount of metal being squeezed per unit of time, which results in an increase in extrusion torque and temperature.

#### 4.2.4. Analysis of Calibration Part

**Figure 16:**
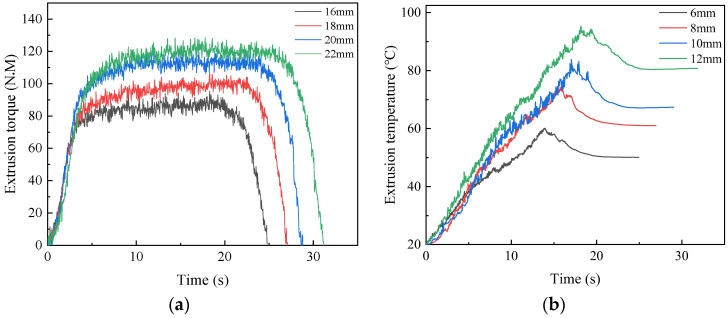
Extrusion torque (**a**) and temperature (**b**)under different calibration part.

The tap models with different calibration parts were established for numerical simulation. The length of calibration part is generally 8 to 11 pitchs, and the simulation is therefore conducted with the calibration part 16 mm, 18 mm, 20 mm and 22 mm, to study the influence of torque and temperature. The curves under different calibration parts are shown in Figure 16a,b.

When the length of calibration part is 16mm, the maximum torque and temperature are 96.03 N·M and 60.08 °C, respectively. As the length of the calibration part increases to 22 mm, the maximum torque and temperature increase to 128.58 N·M and 95.33 °C, respectively. With the increase of the length of calibration part, the contact surface area increases. This leads an increase in the amount of metal being squeezed per unit of time, which results in an increase in extrusion torque and temperature.

#### 4.2.5. Analysis of Extrusion Cone Angle

**Figure 17:**
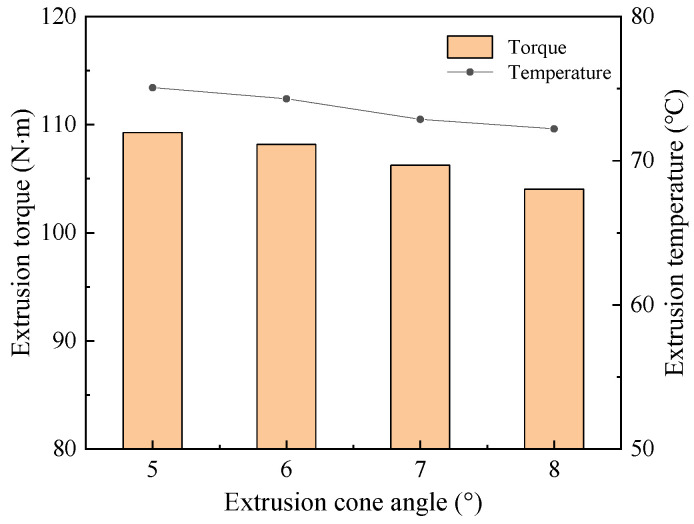
Extrusion torque and temperature under different extrusion cone angles.

According to the production experience of the enterprise, the numerical simulation was conducted with extrusion cone angle 5°, 6°, 7°and 8°. The change under different cone angles is shown in Figure 17, and the maximum torque and temperature are respectively 109.26 N·M and 75.07 °C with extrusion cone angle 5°. As the extrusion cone angle increases to 8°, the maximum torque and temperature increase to 104.03 N·M and 72.21 °C, respectively. The impact of extrusion cone angle on torque and temperature is found to be very weak. Although torque and temperature decrease as extrusion cone angle increase s, the reduction is small. The difference between the maximum torque and the minimum torque under four cone angles is only 4.79%, and the difference between the temperatures is only 3.81%. This is because the change of extrusion cone angle does not have a significant effect on the amount of metal involved in the extrusion. There is no obvious change in the plastic deformation of metal, so the torque and temperature are found to be similar at different cone angles.

### 4.3. Optimization of Tap Structure

#### 4.3.1. Design of Orthogonal Test

Considering that the change in cone angle does not significantly alter the changes in torque and temperature during the forming process, the optimization of this factor is ignored in the orthogonal experiment. The amount of edges, the amount of shovel back, the extrusion cone, and the calibration part of tap are optimized by the orthogonal test, and it is assumed there is no interaction between these four indicators. Table 4 shows the established four-factor four-level table. Table 5 gives the design of the orthogonal test.

#### 4.3.2. Data Analysis of Orthogonal Test

**Figure 18:**
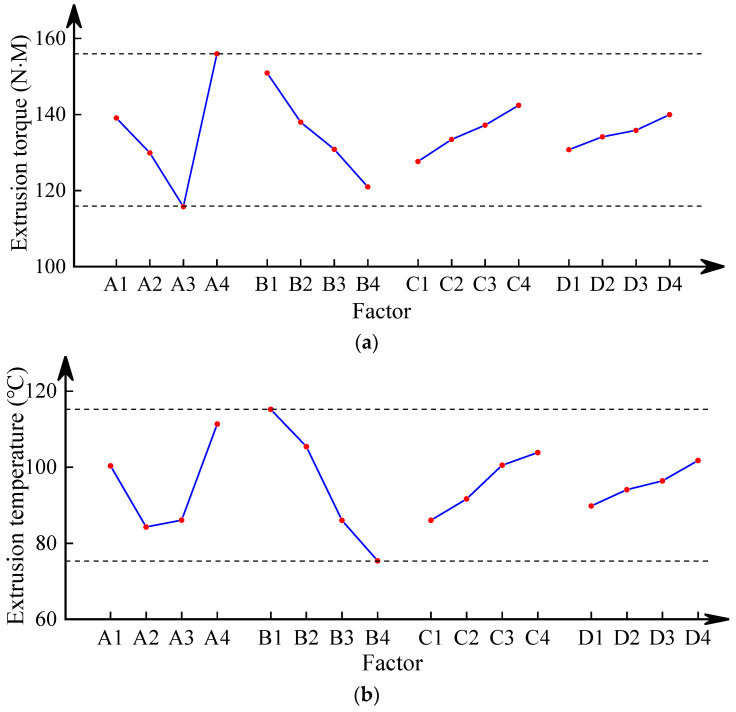
Effect curve of extrusion torque (**a**) and temperature (**b**).

The results based on orthogonal analysis are shown in Table 6, where the *K* reflects the influence of the tap structure on torque and temperature. Torque and temperature are drawn according to the horizontal factor and *K* value, as shown in Figure 18a,b.

In Figure 18, when torque is used as the optimization objective, the impact of backhoe amount is the greatest. The influence order of each factor is as follows: the number of edges > shovel back > extrusion cone > calibration part. For obtaining the smaller torque, according to the effect curve, the best structural parameters of tap are A3B4C1D1—that is, the number of edges is 8, the shovel back is 0.8 mm, the extrusion cone is 6 mm, and the calibration part is 16 mm. Similarly, when temperature is used as the optimization objective, the number of edges and the length of the extrusion cone also have a significant impact, and the order of influence of each factor are: shovel back > the number of edges > extrusion cone > calibration part. The best parameters are as follows: the number of edges is 6, the shovel back is 0.8 mm, the extrusion cone is 6 mm, and the calibration part is 16 mm.

From the above analysis, we can see the optimization schemes obtained under two optimization objectives are not consistent. Among the four influencing factors, the selection of shovel back, extrusion cone and calibration part are the same. But the chosen quantity of edges is not unalike. In referring to the significance of the influence of each factor on the experimental indicators in Table 7, it can be seen that Factor A has a significant impact on the extrusion torque, while Factor A, B, and C have significant effects on the extrusion temperature. However, the sum of the squared deviations of the two indicates that Factor B has a greater impact on the extrusion temperature than Factor A. Choosing A2B4C1D1 level can ensure the minimum extrusion torque and temperature at the same time. The structure parameters of optimized tap are as follows: the number of edges is 8, the shovel back is 0.8 mm, the extrusion cone is 6 mm, and the calibration part is 16 mm.

## 5. Result and Discussion

### 5.1. Extrusion Torque and Temperature

**Figure 19:**
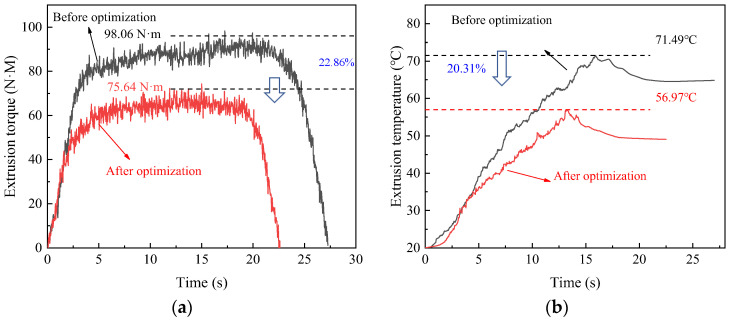
Extrusion torque (**a**) and (**b**) Temperature, before and after optimization.

Figure 19a,b shows the comparison curve before and after optimization. The maximum torque and temperature before optimization are 98.06 N·M and 71.49 °C, respectively. Through optimization, torque and temperature are reduced by 22.86% and 20.31%, respectively. This demonstrates that the optimized parameters may significantly decrease torque and temperature in the machining process, which proves the effectiveness of the tap optimization.

### 5.2. Thread Height

**Figure 20:**
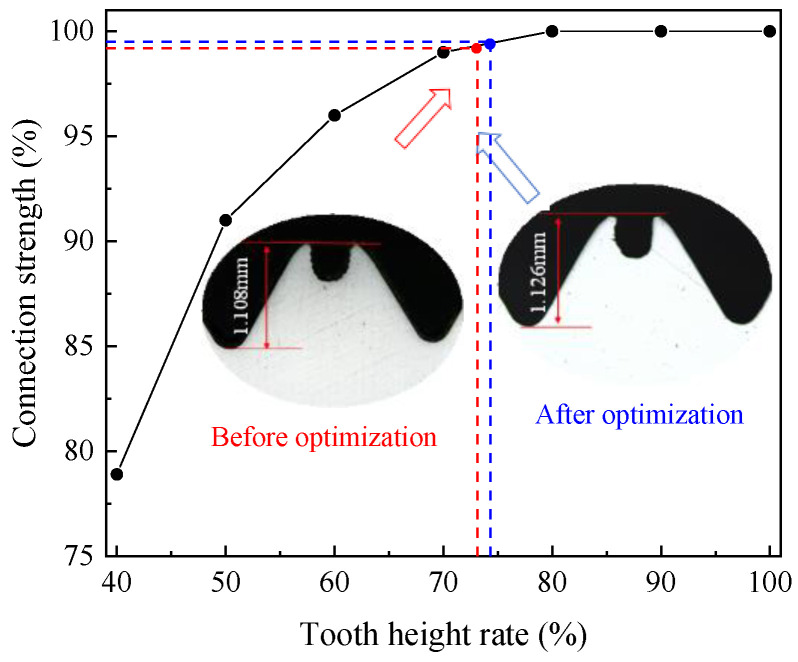
Tooth height, before and after optimization.

The measurement result of thread height before and after optimization is shown in Figure 20. The difference of thread height between before and after optimization is small. The tooth height and tooth height rate of thread are 1.108 mm and 73.14%, respectively. The tooth height and tooth height rate of the optimized thread are 1.126 mm and 74.32%, respectively. This is due to the fact that the essence of the thread-forming process is that the metal gradually fills the groove under extrusion. Tooth height depends on the amount of metal in the deformation zone. Meanwhile, optimized tap does not significantly increase the amount of metal flowing into the groove, meaning that the metal that forms the thread does not increase significantly. As a result, the increase of the thread height is not obvious. The optimized tap can ensure the reduction of torque and temperature, which also proves the effectiveness of the optimization. 

### 5.3. Thread Hardness and Hardened Layer

**Figure 21:**
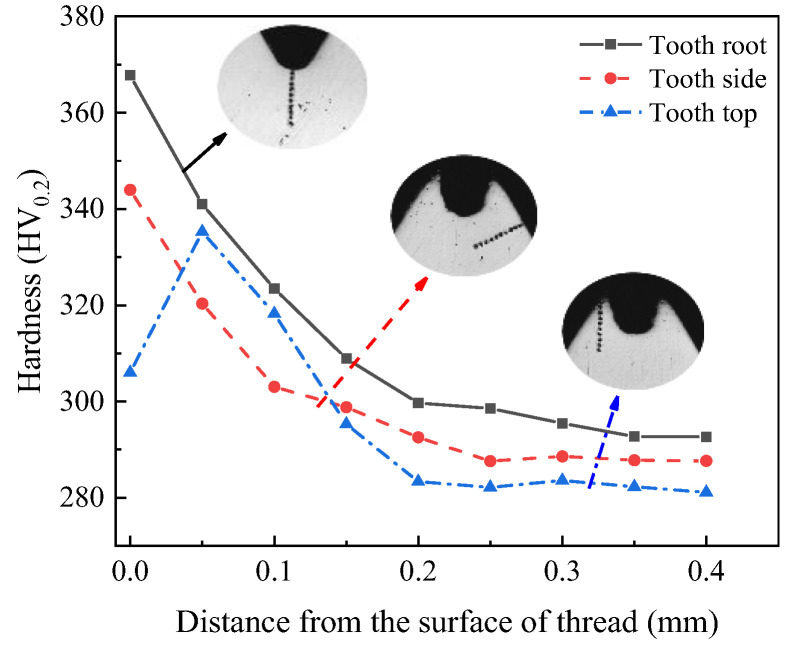
Hardness of optimized thread.

Figure 21 presents the measurement result of hardness of optimized thread. Due to the increase in plastic deformation degree, the optimized surface hardness and hardening depth of the thread have been improved. The hardness and depth of tooth root are 367.74 HV_0.2_ and 0.35 mm, respectively. After optimization, the maximum depth of hardened layer increases by about 0.05 mm, and the maximum increase in tooth root hardness is about 10 HV_0.2_. This indicates that optimized parameters may increase hardness and depth of hardened layer of thread, thus improving the forming quality of thread.

The forming quality of threads was evaluated from the aspects of torque and temperature during cold extrusion processing, thread morphology and microhardness after forming. The results showed that the optimized parameter combination reduced torque by 22.86%, temperature by 20.31%, surface hardness by 2.7%, and hardened layer depth by 14.3%. Although there were no significant improvements in thread morphology and connection strength, it can still effectively improve the quality of thread forming. This validates the effectiveness of tap optimization and forms a high-quality design process.

## 6. Conclusions

The working mechanism of extruded tap is firstly analyzed. The metal of workpiece has plastic deformation under the tap. The metal flows into the groove between tapered teeth. As the tap rotates into the workpiece, depth of tapered teeth extruding into workpiece increases, which makes the metal flowing into the groove increase, and finally form a full thread.

To improve the processing quality of tap, orthogonal optimization was carried out with the edge number, shovel back, extrusion cone, calibration part and extrusion cone angle as factors, and a set of tap structure parameters was obtained to minimize the extrusion torque and temperature. The optimized parameter tap structure is as follows: the number of edges is 8, the shovel back is 0.8 mm, the extrusion cone is 6 mm, and the calibration part is 16 mm. 

When the optimized tap is used for machining, the extrusion torque and the temperature reduced by 22.86% and 20.31%, respectively. The hardness of tooth root increased by 10 HV_0.2_, and the hardness of tooth side increased by 7 HV_0.2_. Although the tooth height increased slightly, it did not reduce the metal flowing into the grooves on the premise of decreasing torque and temperature. The experimental results prove the effectiveness of optimizing the tap, which provides a theoretical basis and technical support for promoting the industrial application of cold extrusion internal thread technology and tool selection. It is worth noting that the lubrication situation during the machining process was limited by the lubrication groove structure of the tap. Subsequent research should improve the existing lubrication groove structure to extend the service life of the tap and further control production costs. 

## Figures and Tables

**Table 1:** Chemical composition of 42CrMo4 high-strength steel.

C	Si	Mn	S	P	Cr	Mo	Fe
0.38	0.3	0.6	0.03	0.03	0.9	0.23	Bal

**Table 2:** Mechanical properties of 42CrMo4 high-strength steel.

Young’s Modulus (GPa)	Poisson’s Ratio	Tensile Strength (MPa)	Yield Strength (MPa)	Elongation (%)
212	0.28	1080	930	12

**Table 3:** Tap structure parameters and simulation parameters.

Type	Parameter Value	Type	Parameter Value
Shovel back/(mm)	0.6	Bottom hole diameter/(mm)	21.20
Extrusion cone angle/(°)	6	Hole depth/(mm)	9
Extrusion cone/(mm)	8	Extrusion speed/(r·min^−1^)	50
Calibration part/(mm)	18	Friction coefficient	0.12
Tap large diameter/(mm)	22.183	Pitch/(mm)	2

**Table 4:** Factor level.

Level	A Edge	B Shovel Back (mm)	C Extrusion Cone (mm)	D Calibration Part (mm)
1	4	0.2	6	16
2	6	0.4	8	18
3	8	0.6	10	20
4	10	0.8	12	22

**Table 5:** Orthogonal design of structural parameters.

Number	A	B (mm)	C (mm)	D (mm)	Empty Column	Torque (N·M)	Temperature (°C)
1	4	0.2	6	16	5	123.25	105.27
2	4	0.4	8	18	6	139.47	105.12
3	4	0.6	10	20	7	137.34	96.88
4	4	0.8	12	22	8	136.24	94.13
5	6	0.2	8	20	8	143.92	100.33
6	6	0.4	6	22	7	129.88	91.02
7	6	0.6	12	16	6	128.68	77.57
8	6	0.8	10	18	5	117.07	68.11
9	8	0.2	10	22	6	138.63	119.23
10	8	0.4	12	20	5	126.87	107.59
11	8	0.6	6	18	8	102.19	65.01
12	8	0.8	8	16	7	95.28	62.48
13	10	0.2	12	18	7	177.84	136.13
14	10	0.4	10	16	8	151.74	134.88
15	10	0.6	8	22	5	155.08	104.71
16	10	0.8	6	20	6	132.24	82.77

**Table 6:** Range analysis results.

Test Index	Factors	*K*_1_	*K*_2_	*K*_3_	*K*_4_	Range Value *R*
Extrusion torque (N·M)	A	134.08	145.91	121.89	124.74	130.57
	B	129.89	136.99	133.44	134.14	134.76
	C	115.74	130.82	136.20	135.09	135.09
	D	154.23	120.21	142.41	139.96	133.52
Extrusion temperature (°C)	A	100.35	115.24	86.02	95.05	96.42
	B	84.26	109.65	93.16	93.59	96.17
	C	88.58	86.04	104.78	96.89	96.63
	D	114.62	76.87	103.86	102.27	98.59

**Table 7:** Variance analysis results.

Test Index	Factors	Sum of Square of Deviations	Free Degree	Mean Square Error	*F*	*P*
Extrusion torque (N·M)	A	3032.94	3	1010.98	59.77	0.0036
	B	1400.19		466.73	27.59	0.0110
	C	885.61		295.20	17.45	0.0211
	D	485.71		161.90	9.57	0.0480
	Error	50.74		16.91		
Extrusion temperature (°C)	A	2220.30	3	740.10	151.21	0.0009
	B	4071.83		1357.28	277.31	0.0004
	C	971.17		323.72	66.14	0.0031
	D	172.86		57.62	11.77	0.0363
	Error	14.68		4.89		

## Data Availability

Data are contained within the article.
